# Unlocking the potential of radiomics in identifying fibrosing and inflammatory patterns in interstitial lung disease

**DOI:** 10.1007/s11547-025-02067-y

**Published:** 2025-08-22

**Authors:** Leonardo Colligiani, Chiara Marzi, Vincenzo Uggenti, Sara Colantonio, Laura Tavanti, Francesco Pistelli, Greta Alì, Emanuele Neri, Chiara Romei

**Affiliations:** 1 Department of Translational Research, Academic Radiology, University of Pisa, 56126 Pisa, Italy; 2 Division of Radiology, Pisa University Hospital, 56126 Pisa, Italy; 3 Department of Statistics, Computer Science, applications “Giuseppe Parenti”, University of Florence, 50134 Florence, Italy; 4 Institute of Information Science and Technologies (ISTI) of the National Research Council (CNR), 56124 Pisa, Italy; 5 Cardiovascular and Thoracic Department, Pisa University Hospital, 56126 Pisa, Italy; 6 Department of Surgical, Medical, Molecular Pathology and Critical Area, University of Pisa, 56126 Pisa, Italy

**Keywords:** Idiopathic pulmonary fibrosis, Interstitial lung disease, Machine learning, Non-specific interstitial pneumonia, Radiomics

## Abstract

**Purpose:**

To differentiate interstitial lung diseases (ILDs) with fibrotic and inflammatory patterns using high-resolution computed tomography (HRCT) and a radiomics-based artificial intelligence (AI) pipeline.

**Materials and methods:**

This single-center study included 84 patients: 50 with idiopathic pulmonary fibrosis (IPF)—representative of fibrotic pattern—and 34 with cellular non-specific interstitial pneumonia (NSIP) secondary to connective tissue disease (CTD)—as an example of mostly inflammatory pattern. For a secondary objective, we analyzed 50 additional patients with COVID-19 pneumonia. We performed semi-automatic segmentation of ILD regions using a deep learning model followed by manual review. From each segmented region, 103 radiomic features were extracted. Classification was performed using an XGBoost model with 1000 bootstrap repetitions and SHapley Additive exPlanations (SHAP) were applied to identify the most predictive features.

**Results:**

The model accurately distinguished a fibrotic ILD pattern from an inflammatory ILD one, achieving an average test set accuracy of 0.91 and AUROC of 0.98. The classification was driven by radiomic features capturing differences in lung morphology, intensity distribution, and textural heterogeneity between the two disease patterns. In differentiating cellular NSIP from COVID-19, the model achieved an average accuracy of 0.89. Inflammatory ILDs exhibited more uniform imaging patterns compared to the greater variability typically observed in viral pneumonia.

**Conclusion:**

Radiomics combined with explainable AI offers promising diagnostic support in distinguishing fibrotic from inflammatory ILD patterns and differentiating inflammatory ILDs from viral pneumonias. This approach could enhance diagnostic precision and provide quantitative support for personalized ILD management.

**Supplementary Information:**

The online version contains supplementary material available at 10.1007/s11547-025-02067-y.

## Introduction

Interstitial lung diseases (ILDs) are a heterogeneous group of conditions affecting the lung parenchyma, caused by systemic diseases, drug, environmental exposures, or of idiopathic origin [1]. Idiopathic pulmonary fibrosis (IPF) and connective tissue diseases (CTDs) are among the most common causes, each with distinct histopathological and radiological patterns.

**Fig. 1:**
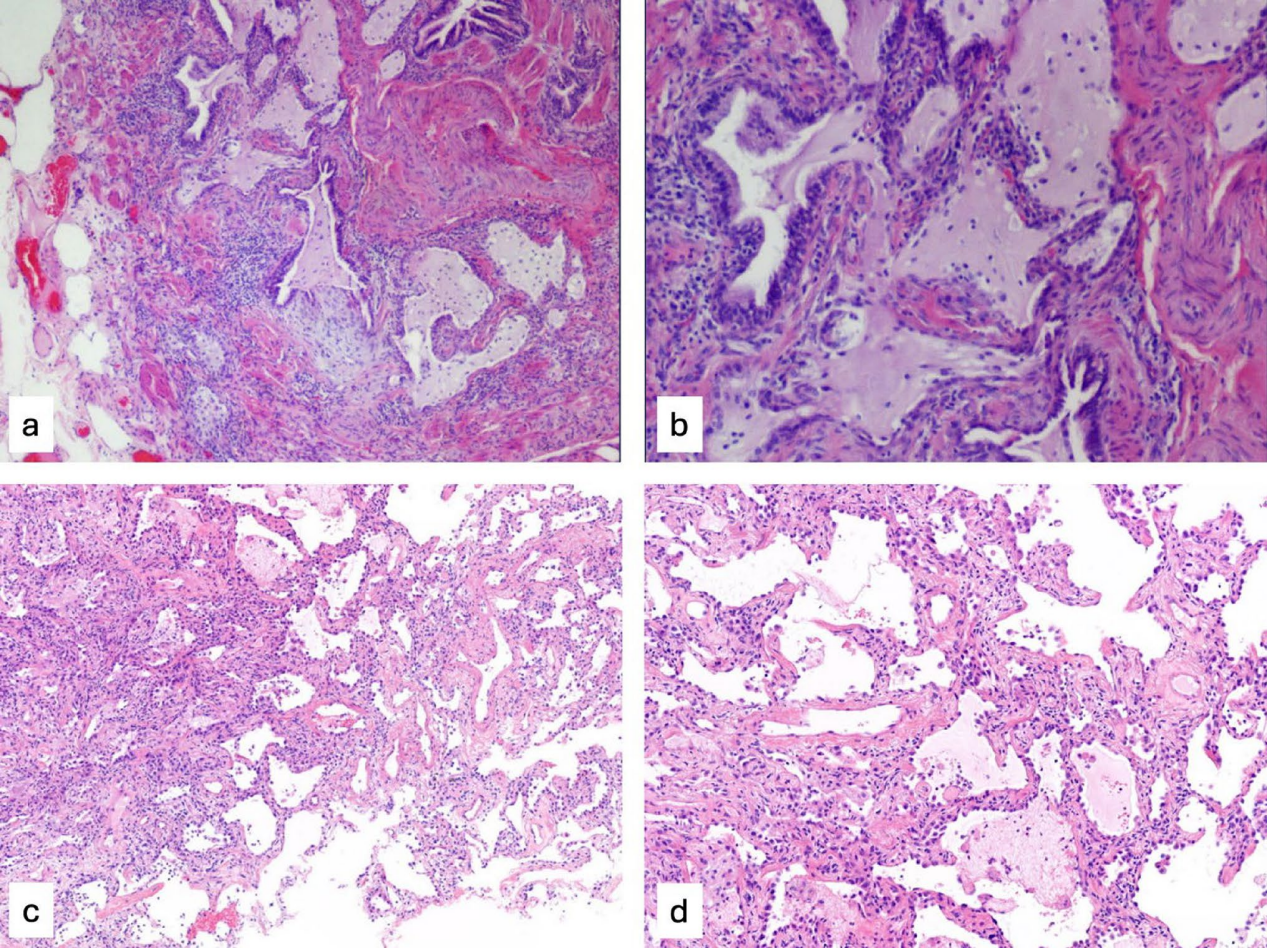
**a**, **b** Histopathologic usual interstitial pneumonia (UIP) pattern. Cryobiopsy of a patient with idiopathic pulmonary fibrosis (IPF) showing foci of microscopic honeycombing associated with dense fibrosis. Microscopic honeycombing cysts are lined by columnar ciliated epithelium and are filled with mucus with inflammatory cells and debris. **c**, **d** Histopathologic non-specific interstitial pneumonia (NSIP) pattern. Cryobiopsy of a patient with cellular NSIP pattern secondary to connective tissue disorder (CTD). A uniform, variable widening of alveolar walls by chronic inflammation and to a lesser extent by dense uniform fibrosis can be seen, with little if any spared alveolar parenchyma (hematoxylin eosin stain, a, c 10X magnification; b, d 20X magnification)

IPF is a severe, progressive fibrotic form of ILD with a global prevalence between 0.33 and 4.51 per 10,000 and incidence of 0.09–0.49 per 10,000, representing a significant health concern [2]. It is radiologically and histologically defined by the usual interstitial pneumonia (UIP) pattern (Fig. 1a and b), featuring honeycombing, traction bronchiectasis, septal thickening, and reduced lung volumes [3]. Antifibrotic therapies like nintedanib and pirfenidone can slow its progression [4].

In contrast, CTDs—systemic autoimmune disorders—commonly lead to ILDs, with prevalence ranges from 11% in rheumatoid arthritis to 56% in mixed CTD, varying by subtype [5]. CTD-related ILDs most often exhibit a non-specific interstitial pneumonia (NSIP) pattern, especially in systemic sclerosis and inflammatory myopathies. UIP is more common in rheumatoid arthritis, while organizing pneumonia pattern is rarer [6]. NSIP ranges from an inflammatory, ground-glass opacity (GGO)-rich cellular form to a fibrotic form with honeycombing and septal thickening (Fig. 1c and d) [7]. Treatment typically involves immunosuppressive or anti-inflammatory agents [8], although some cases still progress [9]. Antifibrotic therapy is now supported in progressive fibrosing ILDs beyond IPF [10, 11].

Accurate ILD diagnosis is essential for guiding treatment—anti-inflammatory vs. antifibrotic—depending on the predominant pattern. Diagnosis should follow multidisciplinary team (MDT) consensus, integrating clinical, functional, radiologic, and, where needed, pathologic data [12].

High-resolution CT (HRCT) is central to diagnosis and often serves as a non-invasive alternative to lung biopsy, enabling identification of suggestive or diagnostic ILD patterns [13]. In contrast, invasive procedures like surgical (SLB) and transbronchial lung biopsy (TBLB) carry risks, including pneumothorax, bleeding, or disease exacerbation [14, 15].

Nonetheless, HRCT interpretation can be challenging, especially in CTD-ILD, where fibrotic features (e.g., honeycombing and septal thickening) may overlap with GGOs. While GGOs often reflect acute inflammation, they may also appear in fibrosis due to preserved airspaces. The overlap with viral pneumonias, such as SARS-CoV-2, further complicates differential diagnosis [16].

In this challenging diagnostic context, radiomics and artificial intelligence (AI) are gaining attention. Radiomics involves the high-throughput extraction of a large number of mathematical features from medical images using advanced algorithms, enabling the identification of structural and textural patterns beyond the perception of the human eye [17–19]. Applied to HRCT, it can help differentiate inflammatory, infectious, and fibrotic patterns, and provide prognostic insights.

Due to the high dimensionality of radiomic data, AI—particularly machine learning (ML)—is essential for uncovering relevant patterns and associations that may be missed by traditional statistics [20, 21]. The integration of explainable AI (XAI) techniques, such as SHapley Additive exPlanations (SHAP) values, improves the transparency and interpretability of machine learning models, by quantifying the contribution of individual features to predictions, thus enhancing clinical interpretability and trust [22].

Given the challenges of overlapping imaging patterns in ILDs and the need for objective diagnostic methods, radiomics merits further integration into ILD workflows. As highlighted by Sica et al. (2025) [23], most prior studies have focused on specific ILD subtypes without directly comparing fibrotic and inflammatory patterns. Our study addresses this gap by developing an AI-based pipeline to distinguish between fibrotic and inflammatory ILDs and to differentiate inflammatory ILDs from viral pneumonia. The pipeline integrates explainable AI techniques to enhance model interpretability and support clinical translation.

## Material and methods

### Participants

This cross sectional, single-center study was approved by the institutional review board, and written informed consent was obtained from all participants.

For the primary objective of this study, we analyzed two groups of patients based on their MDT diagnoses: patients with cellular NSIP secondary to CTD, representing a predominantly inflammatory pattern, and patients with IPF, reflecting a purely fibrotic pattern. For the secondary objective, we included a third group of patients diagnosed with COVID-19 pneumonia, serving as an example of viral diffuse lung disease.

Thirty-four cellular NSIP patients were selected from the ILD database of Pisa University Hospital, which includes 1302 patients evaluated by the MDT for ILDs. HRCT scans of all patients diagnosed with NSIP secondary to CTD were reviewed by a thoracic radiologist with over 10 years of experience in ILD imaging (CR). We included only cases with extensive radiological findings consistent with a cellular NSIP pattern, as defined by established imaging criteria. For the IPF group, we randomly selected 50 patients diagnosed with IPF from the same ILD database. HRCT scans were independently reviewed to include only those meeting the criteria for a definite UIP pattern, ensuring the representation of a homogeneous fibrotic phenotype.

Fifty COVID-19 patients were randomly selected from a database of 136 COVID-19 cases admitted to the emergency department at Pisa University Hospital between March 2020 and December 2022. All patients had a confirmed positive SARS-CoV-2 RT-PCR test. HRCT scans were reviewed to include only those with extensive GGO as the predominant feature of lung involvement, representing the typical radiological pattern of COVID-19 pneumonia.

Across all groups, additional inclusion criteria included the availability of HRCT scans acquired with adequate technical parameters, as detailed in the Chest CT protocols section. Exclusion criteria were the presence of image-degrading artifacts (e.g., motion artifacts and beam hardening) and cases with minimal radiologic lung abnormalities insufficient for radiomic analysis.

**Fig. 2:**
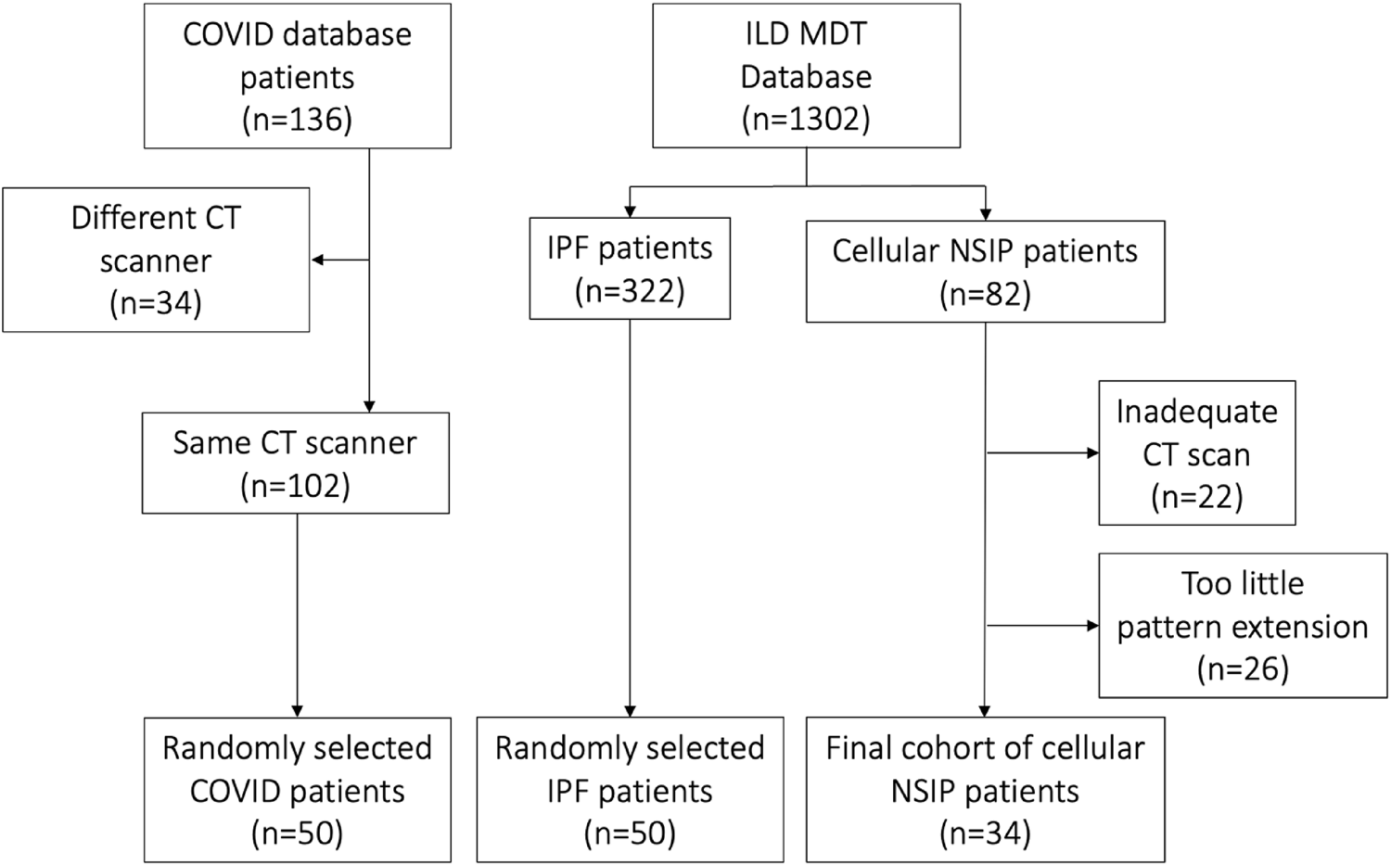
Flowchart of patient selection process for each group. Random selection for IPF and COVID patients was performed by simple random sampling using the *sample()* function in RStudio, with a fixed seed (set to 42) to ensure reproducibility and no stratification. ILD: interstitial lung disease; IPF: interstitial pulmonary fibrosis; NSIP: non-specific interstitial pneumonia

The final cohort consisted of 34 patients with cellular NSIP, 50 patients with IPF, and 50 patients with COVID-19 pneumonia. The patient selection process for each group is illustrated in Fig. 2.

### Chest CT protocols

All non-enhanced chest HRCT examinations were performed during a single full inspiratory breath-hold with patients in the supine position, covering the entire lung field (from just above the lungs apex to just below the costophrenic angles).

For patients with IPF and cellular NSIP, CT scans were acquired using a 64-slice Siemens Somatom Sensation scanner (Siemens Healthineers) with the following technical parameters: 120 kV, 250 mAs, collimation width of 0.6 mm, matrix size of 512 × 512, spiral pitch factor of 1.0, tube rotation time of 0.8 s/rotation and a reconstruction thickness of 1.5 mm. Images were reconstructed using the B60, B31, and B35 kernels to optimize both lung parenchyma and mediastinal window evaluation, but only the sharpest kernel (i.e., B60) was used for all the steps relative to this work (visual analysis, image segmentation and radiomic features extraction).

For COVID-19 patients, CT scans were performed using a 40-slice Siemens Somatom Sensation scanner (Siemens Healthineers) with the following parameters: 120 kV, 284 mAs, collimation width of 0.6 mm, matrix size of 512 × 512, spiral pitch factor of 1.84, tube rotation time of 0.8 s/rotation and a reconstruction thickness of 1.5 mm. Images were reconstructed using the B31 and B70 kernels, optimized for lung parenchyma and mediastinal assessment, but only the sharpest kernel (i.e., B70) was used for all the steps relative to this work (visual analysis, image segmentation and radiomic features extraction).

### Image processing and features extraction

**Fig. 3:**
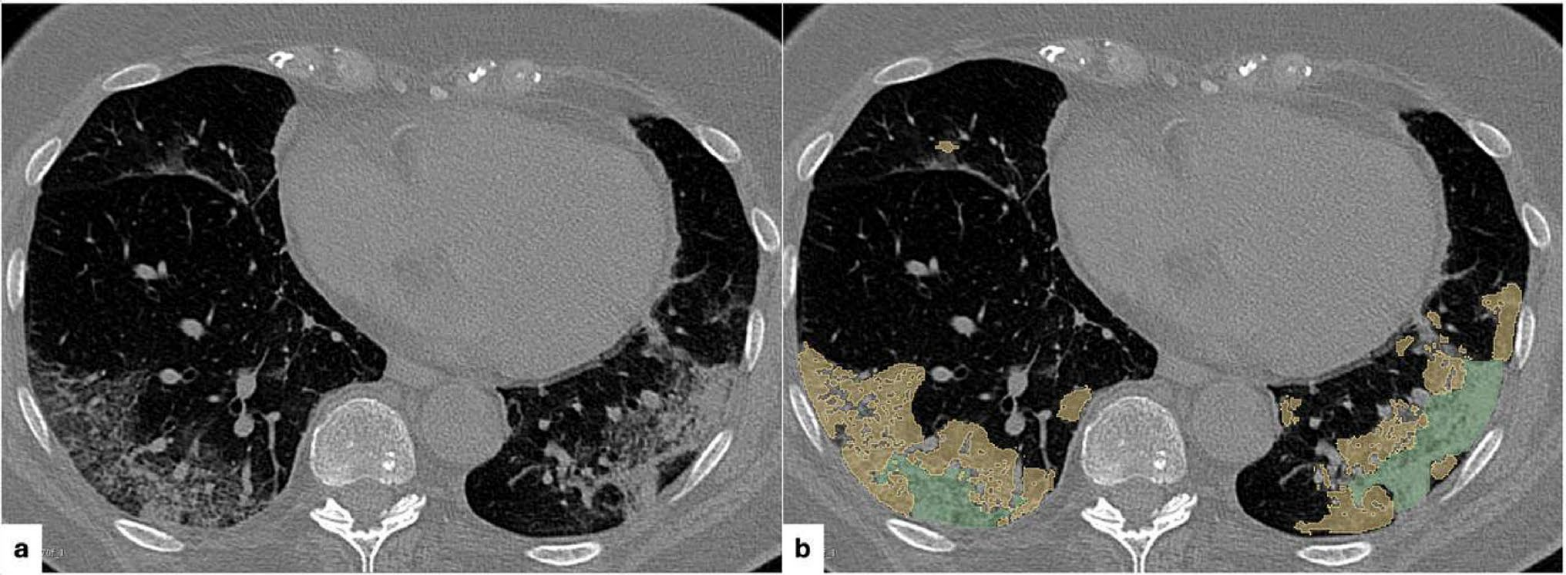
Representative axial CT slice **a** and corresponding automatic segmentation mask **b** for a patient with COVID-19 pneumonia. The segmentation, generated by our deep learning model, highlights in yellow the regions of interstitial involvement and in green the areas of consolidation (the latter not included in the present analysis)

We applied a semi-automatic two-step segmentation process to each CT image. In the first step, an in-house deep learning network, specifically trained to recognize and delineate ILD regions, performed the initial segmentation of the entire lung volume [24]. In the second step, each segmentation was manually reviewed in consensus by a thoracic radiologist (CR) with over 10 years of experience in thoracic imaging and a fourth-year senior radiology resident (LC) (we reported a 2-D example of ILD segmentation in a COVID-19 patient in Fig. 3). This review ensured the accuracy of the segmentation, and any errors or inaccuracies were corrected using 3D Slicer (version 5.6.2 for macOS, http://www.slicer.org), an open-source software for medical image visualization and analysis [25].

To ensure spatial consistency across patients, all CT images and segmentations were resampled to an in-plane resolution of 0.6 mm^2^ using third-order B-spline interpolation. No intensity normalization was applied, as CT values are expressed in HU, which are already standardized and physically meaningful.

From each segmented region of interest (ROI)—single, unified ROI, even if anatomically disconnected, for each patient—we extracted a total of 103 radiomic features. These included 17 three-dimensional (3D) shape features, 18 first-order statistical features, and 68 second-order textural features. The 3D shape features encompassed conventional descriptors such as elongation, flatness, volume, and sphericity, along with fractal descriptors designed to capture the morphological complexity of ILD regions [26–33]. The fractal features were computed using an enhanced 3D box-counting method with automated selection of the optimal scaling interval based on the adjusted R^2^ of the log–log regression, as proposed in [29] and implemented in the open-source Fractalbrain Toolkit (version 1.0) [34]. The first-order features included 16 intensity-based statistical metrics, along with two intensity histogram features: entropy and uniformity, which quantify the distribution and randomness of voxel intensities within the ROI.

The second-order textural features were derived from five different matrices that capture spatial relationships between voxel intensities. These matrices included the gray-level co-occurrence matrix (GLCM), from which 22 features were extracted; the gray-level run-length matrix (GLRLM), with 16 extracted features; the gray-level size zone matrix (GLSZM), also contributing 16 features; and the gray-level dependence matrix (GLDM), from which 14 features were extracted using a coarseness parameter set to $\alpha =0$. The complete list of extracted radiomic features is provided in Supplementary Table S1.

Radiomic feature extraction was performed using PyRadiomics (version 3.0.1) [35] (https://pyradiomics.readthedocs.io/en/v3.0.1/index.html). All radiomic features were computed in accordance with the definitions provided by the Image Biomarker Standardization Initiative (IBSI) [36], except for specific features detailed in Supplementary Table S1.

### Machine learning analysis

We performed a binary classification to distinguish IPF and NSIP using an eXtreme Gradient Boosting (XGBoost) method (version 1.5.0). We used the default hyperparameters, including *binary:logistic* as the objective function. XGBoost is a scalable and efficient tree-boosting system that has consistently demonstrated state-of-the-art performance in medical imaging tasks involving high-dimensional, nonlinear associations, and is compatible with explainable AI techniques such as SHAP [37–39].

To train and test the classifier, we implemented a 1000-times repeated stratified bootstrapping procedure. In each iteration, we generated a training set by sampling with replacement from the original dataset (with a fixed seed to ensure reproducibility), ensuring that the training set matched the size of the full dataset. The test set consisted of the unique instances excluded from the training set, i.e., the out-of-bag samples. By employing bootstrap sampling, we maintain the statistical assumptions required for robust inference [40–44], ensuring that the results are more reliable and generalizable, particularly in the context of medical applications where robustness is essential.

For final performance metrics, we calculated the mean accuracy, balanced accuracy, average precision, and area under the Receiver Operating Characteristic (ROC) curve and their 95% bootstrap intervals (BIs) across the 1000 test sets. Additionally, we computed the average confusion matrix, normalized by the number of patients in each actual class (50 samples for the IPF group, and 34 samples for the NSIP group), and reported its corresponding 95% BIs.

To assess the overall contribution of each feature to class predictions, we applied SHapley Additive exPlanations (SHAP) values, an explainable AI technique that quantifies the importance of each feature in determining the model’s output [45]. Each SHAP value represents a real number associated with a particular feature of an individual sample (i.e., a subject). To obtain the feature contributions, SHAP values were averaged, in absolute value, across patients.

We used the same machine learning framework for identifying NSIP vs. COVID-19 patients.

The full codebase supporting this machine learning analysis is publicly available at https://github.com/chiaramarzi/radiomics-ild-patterns [46].

## Results

### Cohort description

A total of 84 patients were included in the study, with 50 individuals in the IPF group and 34 in the cellular NSIP group. The demographic and clinical characteristics of the cohort are summarized in Table 1. Notably, half of the patients were male, with a higher proportion in the IPF group. The majority of participants (92.4%) were non-smokers, with 45.6% having never smoked and 46.8% being former smokers. At spirometry, IPF patients showed mildly impaired FVC (average 76.02%), and moderate reduction in DLCO values (average 43.44%).

**Table 1:** Demographic and clinical characteristics of the study cohort

	Overall	NSIP	IPF
N	84	34	50
Sex (males), n (%)	42 (50)	9 (26.5)	33 (66.0)
Age (years), average (SD)	67.94 (11.85)	59.88 (12.94)	73.42 (7.04)
Smoke, n (%)			
Never	36 (45.6)	19 (61.3)	17 (35.4)
Current	6 (7.6)	6 (19.4)	0 (0.0)
Former	37 (46.8)	6 (19.4)	31 (64.6)
FVC (% predicted), average (SD)	82.03 (20.65)	94.90 (18.50)	76.02 (18.93)
DLCO (% predicted), average (SD)	51.95 (20.31)	68.57 (21.78)	43.44 (13.13)

DLCO: diffusion lung carbon monoxide; FVC: forced vital capacity; IPF: idiopathic pulmonary fibrosis; NSIP: Non−specific interstitial pneumonia; SD: standard deviation

### IPF vs. NSIP

The primary objective of this study was to evaluate the classifier’s ability to distinguish between IPF and NSIP based on radiomic features. The average performance metrics of the classifier are reported in Table 2. Overall, all metrics indicate that the model performed reasonably well in distinguishing between IPF and NSIP across the bootstrap-derived test sets (AUROC = 0.98, 95% BI [0.91, 1.00]).

**Table 2:** NSIP vs. IPF: average and 95% BI performances of the classifier over the 1000 bootstrap-derived test sets

	Training set	Test set
Accuracy	1.00 [1.00, 1.00]	0.91 [0.79, 1.00]
Balanced accuracy	1.00 [1.00, 1.00]	0.91 [0.79, 1.00]
Average precision	1.00 [1.00, 1.00]	0.96 [0.87, 1.00]
AUROC	1.00 [1.00, 1.00]	0.98 [0.91, 1.00]

AUROC: area under the ROC curve

**Fig. 4:**
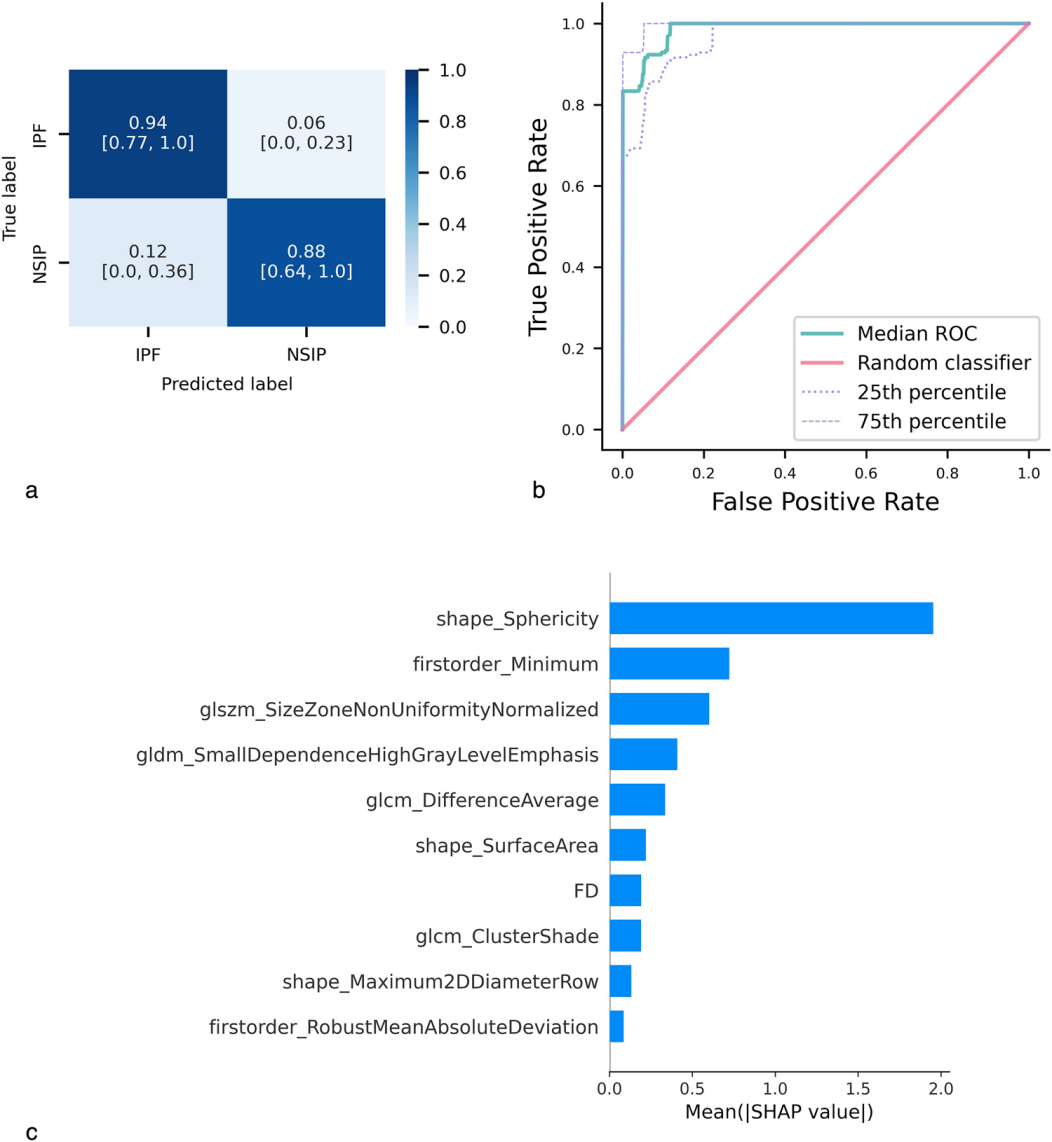
Classification of IPF vs. NSIP groups. **a** Average confusion matrix, normalized by the number of patients in the actual classes, with 95% bootstrap intervals (BIs), for the NSIP vs. IPF classification. **b** Median ROC curve over the 1000 bootstrap repetitions, along with the 25th and 75th percentile curves. **c** Average absolute SHAP values of the ten most influential radiomic features in predicting IPF vs. NSIP

The detailed classification performance is presented in the average confusion matrix and ROC curve shown in Fig. 4a and b. The average confusion matrix includes normalized values and their respective 95% confidence intervals (CIs). The confusion matrix highlights the classifier’s ability to correctly identify each group, with a percentage of correctly classified subjects equal to or greater than 88%. Regarding misclassification patterns, we observed that ILD regions from IPF patients were misclassified as NSIP in 6% of cases.

The overall average SHAP values for each feature, as illustrated in Fig. 4c, reveal the relative importance of radiomic features in distinguishing between the two groups. Specifically, ILD regions from IPF patients appear to exhibit less spherical shapes, the presence of small areas with high voxel density, and well-defined regions with high local contrast—indicating less homogeneity compared to ILD regions from NSIP patients.

### NSIP vs. COVID-19

To further explore the clinical utility of radiomic features in ILD differentiation, we conducted a secondary analysis to assess the classifier’s performance in distinguishing NSIP from COVID-19. The classifier’s average performance metrics are summarized in Table 3. Overall, the results demonstrate that the model achieved good performance in differentiating between NSIP and COVID-19 across the bootstrap-derived test sets (AUROC = 0.96, 95% BI [0.94, 0.99]).

**Table 3:** NSIP s. COVID-19: average and 95% BI performances of the classifier over the 1000 bootstrap-derived test sets

	Training set	Test set
Accuracy	1.00 [1.00, 1.00]	0.89 [0.77, 1.00]
Balanced accuracy	1.00 [1.00, 1.00]	0.88 [0.76, 1.00]
Average precision	1.00 [1.00, 1.00]	0.95 [0.85, 1.00]
AUROC	1.00 [1.00, 1.00]	0.96 [0.94, 0.99]

AUROC: area under the ROC curve

**Fig. 5:**
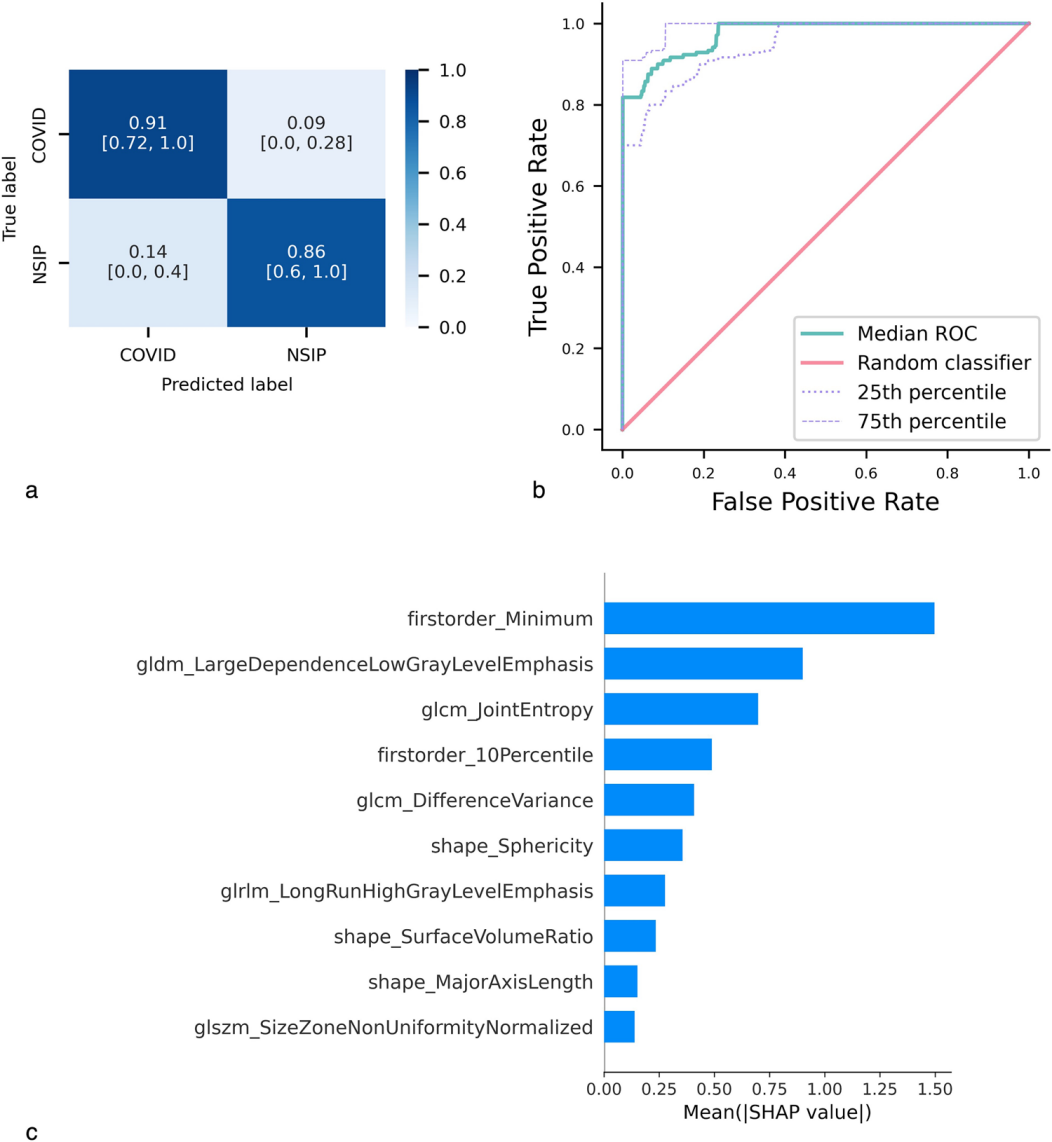
Classification of NSIP vs. COVID-19 groups. **a** Average confusion matrix, normalized by the number of patients in the actual classes, with 95% bootstrap intervals (BIs), for the NSIP vs. COVID-19 classification. **b** Median ROC curve over the 1000 bootstrap repetitions, along with the 25th and 75th percentile curves. **c** Average absolute SHAP values of the ten most influential radiomic features in predicting NSIP vs. COVID-19

A detailed analysis of the classification performance is provided in the average confusion matrix and ROC curve in Fig. 5a and b. The average confusion matrix reports normalized values alongside their 95% confidence intervals (CIs). The matrix showcases the model’s effectiveness in correctly identifying each group, with a classification accuracy of 86% or higher. However, we observed some misclassification patterns, with 14% of ILD regions from NSIP patients being incorrectly classified as COVID.

The SHAP value analysis, shown in Fig. 5c, highlights the relative importance of radiomic features in distinguishing the two groups. Texture-related features emerged as key drivers of the model’s predictions, underscoring the relevance of local intensity patterns in distinguishing between the two groups. In particular, ILD regions from NSIP patients exhibited a more homogeneous and organized texture profile compared to those from COVID-19 patients, which were characterized by greater heterogeneity and less regularity in their radiomic patterns.

## Discussion

This study highlights the potential of a radiomics-based pipeline, combining semi-automatic CT segmentation and machine learning, to differentiate fibrotic ILD patterns from inflammatory ones, as well as differentiating inflammatory ILDs from viral pneumonia.

Our model demonstrated strong performance in differentiating IPF from cellular NSIP, with a test set accuracy of 0.89 and an AUROC of 0.96. These results are in line with Chen et al. (2023) [47], though their slightly different metrics likely reflect the inclusion of both fibrotic and cellular NSIP patients, introducing additional heterogeneity.

Key features distinguishing IPF from NSIP included less spherical lesion shapes, small high-intensity areas, well-defined regions with high local contrast, and greater heterogeneity in ILD-affected regions in IPF compared to cellular NSIP. These features were highlighted by SHAP values as key drivers of the model’s predictions. While our interpretation of these features is consistent with the known histopathological differences between IPF and NSIP, we acknowledge that SHAP values reflect model behavior and do not inherently imply direct biological significance. In IPF, fibrosis typically presents as patchy areas sharply demarcated from normal lung, with fibroblastic foci and dense spatial and temporal heterogeneity, architectural distortion, and honeycombing (Fig. 1a and b). In contrast, NSIP, especially in its cellular form, shows more uniform inflammation with minimal fibrosis and preserved parenchymal architecture (Fig. 1c and d) [48].

Our hypothesis—that these radiomic patterns reflect compact fibrotic foci and architectural distortion in IPF, and more uniform inflammatory involvement in NSIP—is further supported by Haga et al. [49], who correlated radiomic features with histologically assessed inflammatory cellularity. NSIP cases with high inflammatory burden exhibited uniformly low CT values. This convergence, despite methodological differences, reinforces confidence in the radiomic-pathology link. Still, further biological validation is warranted.

From a methodological perspective, this study underscores the advantages of machine learning in leveraging high-dimensional radiomic features, overcoming the limitations of conventional statistical methods. The adoption of a stratified sampling approach ensured that the distribution of patient groups (IPF, NSIP, and COVID-19) in the training and test sets reflected their proportions in the overall dataset, minimizing sampling bias and enhancing classification robustness. To improve reliability, we performed 1000 bootstrap iterations for data splitting, which helped reduce model dependence on specific training data partitions, minimize variance in performance estimation, and maintain minimal bias, consistent with the recommendations by Molinaro et al., [50] and Kim [51]. The adopted validation scheme also aimed to mitigate the limitation of the relatively small sample size—particularly in the NSIP group. This approach enhances the robustness of performance estimates despite data constraints. Nevertheless, future studies involving larger and more heterogeneous patient populations will be crucial to further validate and expand upon these results. Additionally, the use of bootstrap resampling allowed for the calculation of plausible performance intervals, providing a more reliable and interpretable assessment of model performance.

Another key methodological strength is the integration of XAI techniques, specifically SHAP values estimation, which allowed us to interpret the impact of specific radiomic features on model predictions. This approach bridges the gap between quantitative radiomic analysis and traditional radiological interpretation, enhancing the interpretability of the results and fostering a stronger connection between computational and clinical insights.

Regarding our secondary aim, the radiomics-based model successfully differentiated cellular NSIP from COVID-19 pneumonia, achieving high accuracy and AUROC in the test set. The primary distinguishing factors were texture-related features, with cellular NSIP demonstrating greater homogeneity compared to COVID-19. Few studies have explored the role of radiomics in distinguishing GGO patterns across different pulmonary conditions. Delli Pizzi and colleagues demonstrated that a radiomics-based machine learning model could effectively differentiate COVID-19-related GGO from those associated with other acute lung diseases, achieving an AUROC of 0.868 [52]. Their findings suggested that COVID-19-associated GGO exhibits a more homogeneous texture compared to other etiologies. The discrepancy between their results and ours may be attributed to differences in segmentation strategies. While Delli Pizzi et al. focused solely on GGO regions, in our study, we segmented all lung regions affected by COVID-19, including areas with GGO and crazy paving patterns. This broader segmentation approach likely resulted in more heterogeneous textural patterns for COVID-19 compared to cellular NSIP, where GGO predominates.

These promising findings provide a strong foundation for future research aimed at further refining and expanding the clinical application of radiomics. In particular, we plan to investigate the spatial distribution and longitudinal evolution of significant radiomic features in the follow-up of patients with varying degrees of combined fibrosis and inflammation. This will allow for a more precise assessment of disease progression and the potential transition of ILD toward a more fibrotic phenotype. Such insights could assist clinicians in determining the optimal timing for transitioning from anti-inflammatory to antifibrotic therapy, ultimately leading to personalized treatment strategies and improved patient outcomes.

This study has several limitations. First, it is a single-center study that relies on retrospective data, which may limit the generalizability of the findings to other institutions and populations. Second, the relatively small patient cohort reduces statistical power and constrains the ability to optimize the model’s hyperparameters effectively. A larger dataset could have allowed for more robust model evaluation and fine-tuning, further improving performance while minimizing overfitting and preserving generalizability. Third, the classification of fibrotic and inflammatory patterns was based on HRCT imaging and multidisciplinary consensus, without histopathologic confirmation. While multidisciplinary assessment is a widely accepted standard in ILD diagnosis, the absence of pathological validation introduces an element of uncertainty, as imaging findings alone may not fully capture the underlying biological processes. Regarding the secondary aim, the NSIP and COVID-19 groups were acquired using different CT scanners and protocols, and due to the complete overlap between scanner type and disease group, no harmonization technique could be applied without risking the removal of disease-specific radiomic information [53, 54].

## Conclusion

This study demonstrated the potential of a radiomics-based model in distinguishing ILDs with predominantly fibrotic patterns from those with predominantly inflammatory patterns, as well as in differentiating inflammatory ILDs from viral pneumonias. By integrating semi-automatic segmentation and advanced radiomic feature analysis, this approach underscores the value of quantitative imaging in overcoming diagnostic challenges in ILDs and related conditions.

## Supplementary Information

Below is the link to the electronic supplementary material.

Supplementary file1 (DOCX 2146 KB)
