# Supplementary material for: Unlocking the potential of radiomics in identifying fibrosing and inflammatory patterns in interstitial lung disease
Source: Radiol Med. 2025 Aug 22;130(11):1797–807. doi: 10.1007/s11547-025-02067-y (PMC12605480; doi:10.1007/s11547-025-02067-y)
Supplement: Supplementary file 1 — Supplementary file1 (DOCX 2146 KB) [file 11547_2025_2067_MOESM1_ESM.docx]

**Supplementary Information**

**Unlocking the Potential of Radiomics in Identifying Fibrosing and Inflammatory Patterns in Interstitial Lung Disease**

**Supplementary Table S1. Names and explanations of the top-ranking radiomic features distinguishing IPF from NSIP, and NSIP from COVID-19.**

| **#** | **Radiomic feature name** | **Explanation** |
| --- | --- | --- |
| 1 | shape_Elongation |  |
| 2 | shape_Flatness |  |
| 3 | shape_LeastAxisLength |  |
| 4 | shape_MajorAxisLength |  |
| 5 | shape_Maximum2DDiameterColumn |  |
| 6 | shape_Maximum2DDiameterRow |  |
| 7 | shape_Maximum2DDiameterSlice |  |
| 8 | shape_Maximum3DDiameter |  |
| 9 | shape_MeshVolume |  |
| 10 | shape_MinorAxisLength |  |
| 11 | shape_Sphericity | It measures ow closely the shape of a region resembles a perfect sphere; lower values indicate more irregular or distorted morphology. |
| 12 | shape_SurfaceArea |  |
| 13 | shape_SurfaceVolumeRatio |  |
| 14 | shape_VoxelVolume |  |
| 15 | firstorder_10Percentile |  |
| 16 | firstorder_90Percentile |  |
| 17 | firstorder_Energy* |  |
| 18 | firstorder_Entropy |  |
| 19 | firstorder_InterquartileRange |  |
| 20 | firstorder_Kurtosis* |  |
| 21 | firstorder_Maximum |  |
| 22 | firstorder_MeanAbsoluteDeviation |  |
| 23 | firstorder_Mean |  |
| 24 | firstorder_Median |  |
| 25 | firstorder_Minimum | The smallest gray-level intensity value within the Region of Interest (ROI). |
| 26 | firstorder_Range |  |
| 27 | firstorder_RobustMeanAbsoluteDeviation |  |
| 28 | firstorder_RootMeanSquared* |  |
| 29 | firstorder_Skewness |  |
| 30 | firstorder_TotalEnergy* |  |
| 31 | firstorder_Uniformity |  |
| 32 | firstorder_Variance |  |
| 33 | glcm_Autocorrelation |  |
| 34 | glcm_JointAverage |  |
| 35 | glcm_ClusterProminence |  |
| 36 | glcm_ClusterShade |  |
| 37 | glcm_ClusterTendency |  |
| 38 | glcm_Contrast |  |
| 39 | glcm_Correlation |  |
| 40 | glcm_DifferenceAverage |  |
| 41 | glcm_DifferenceEntropy |  |
| 42 | glcm_DifferenceVariance |  |
| 43 | glcm_JointEnergy |  |
| 44 | glcm_JointEntropy |  |
| 45 | glcm_Imc1 |  |
| 46 | glcm_Imc2 |  |
| 47 | glcm_Idm |  |
| 48 | glcm_Idmn |  |
| 49 | glcm_Id |  |
| 50 | glcm_Idn |  |
| 51 | glcm_InverseVariance |  |
| 52 | glcm_MaximumProbability |  |
| 53 | glcm_SumEntropy |  |
| 54 | glcm_SumSquares |  |
| 55 | glrlm_GrayLevelNonUniformity |  |
| 56 | glrlm_GrayLevelNonUniformityNormalized |  |
| 57 | glrlm_GrayLevelVariance |  |
| 58 | glrlm_HighGrayLevelRunEmphasis |  |
| 59 | glrlm_LongRunEmphasis |  |
| 60 | glrlm_LongRunHighGrayLevelEmphasis |  |
| 61 | glrlm_LongRunLowGrayLevelEmphasis |  |
| 62 | glrlm_LowGrayLevelRunEmphasis |  |
| 63 | glrlm_RunEntropy |  |
| 64 | glrlm_RunLengthNonUniformity |  |
| 65 | glrlm_RunLengthNonUniformityNormalized |  |
| 66 | glrlm_RunPercentage |  |
| 67 | glrlm_RunVariance |  |
| 68 | glrlm_ShortRunEmphasis |  |
| 69 | glrlm_ShortRunHighGrayLevelEmphasis |  |
| 70 | glrlm_ShortRunLowGrayLevelEmphasis |  |
| 71 | glszm_GrayLevelNonUniformity |  |
| 72 | glszm_GrayLevelNonUniformityNormalized |  |
| 73 | glszm_GrayLevelVariance |  |
| 74 | glszm_HighGrayLevelZoneEmphasis |  |
| 75 | glszm_LargeAreaEmphasis |  |
| 76 | glszm_LargeAreaHighGrayLevelEmphasis |  |
| 77 | glszm_LargeAreaLowGrayLevelEmphasis |  |
| 78 | glszm_LowGrayLevelZoneEmphasis |  |
| 79 | glszm_SizeZoneNonUniformity |  |
| 80 | glszm_SizeZoneNonUniformityNormalized | Measures the variability of size zone volumes (i.e., the number of connected voxels that share the same intensity) across the image, normalized by the total number of zones. In lung CTs, a higher value may indicate a more heterogeneous parenchymal texture. |
| 81 | glszm_SmallAreaEmphasis |  |
| 82 | glszm_SmallAreaHighGrayLevelEmphasis |  |
| 83 | glszm_SmallAreaLowGrayLevelEmphasis |  |
| 84 | glszm_ZoneEntropy |  |
| 85 | glszm_ZonePercentage |  |
| 86 | glszm_ZoneVariance |  |
| 87 | gldm_DependenceEntropy |  |
| 88 | gldm_DependenceNonUniformity |  |
| 89 | gldm_DependenceNonUniformityNormalized |  |
| 90 | gldm_DependenceVariance |  |
| 91 | gldm_GrayLevelNonUniformity |  |
| 92 | gldm_GrayLevelVariance |  |
| 93 | gldm_HighGrayLevelEmphasis |  |
| 94 | gldm_LargeDependenceEmphasis |  |
| 95 | gldm_LargeDependenceHighGrayLevelEmphasis |  |
| 96 | gldm_LargeDependenceLowGrayLevelEmphasis |  |
| 97 | gldm_LowGrayLevelEmphasis |  |
| 98 | gldm_SmallDependenceEmphasis |  |
| 99 | gldm_SmallDependenceHighGrayLevelEmphasis | Measures the joint occurrence of small dependencies (i.e., groups of neighboring voxels with similar intensity) and high gray-level values within the ROI. |
| 100 | gldm_SmallDependenceLowGrayLevelEmphasis |  |
| 101 | mfs* |  |
| 102 | Mfs* |  |
| 103 | FD* |  |

FD: fractal dimension; glcm: gray-level co-occurrence matrix; gldm: gray-level dependence matrix; glrlm: gray-level run-length matrix; glszm: gray-level size zone matrix; mfs: minimum fractal scale; Mfs: maximum fractal scale.

*Features which did not adhere to IBSI definitions (<https://pyradiomics.readthedocs.io/en/v3.0.1/features.html>)
